# *Brucella abortus* Strain RB51 Vaccine: Immune Response after Calfhood Vaccination and Field Investigation in Italian Cattle Population

**DOI:** 10.1155/2008/584624

**Published:** 2008-04-09

**Authors:** Manuela Tittarelli, Barbara Bonfini, Fabrizio De Massis, Armando Giovannini, Mauro Di Ventura, Donatella Nannini, Vincenzo Caporale

**Affiliations:** ^1^Istituto Zooprofilattico Sperimentale dell'Abruzzo e del Molise “G. Caporale”, Campo Boario, 64100 Teramo, Italy; ^2^Department of Animal health and Welfare (AHAW) Unit, European Food Safety Authority (EFSA), 43100 Parma, Italy

## Abstract

Immune response to *Brucella abortus* strain RB51 vaccine was measured in cattle vaccinated at calfhood. After an increase at day 6 post-vaccination (pv), the antibody level recorded in the 10 vaccinated animals remained constant for two months, and then progressively decreased. All vaccinated animals remained negative from day 162 pv to the end of the study (day 300 pv). Only at days 13 and 14 pv the RB51-CFT showed 100% sensitivity (credibility interval (CI) 76.2%–100%). The results indicate that the possibility to use RB51-CFT for the identification of cattle vaccinated at calfhood with RB51 is limited in time. A field investigation was carried out on 26,975 sera collected on regional basis from the Italian cattle population. The study outcomes indicate that in case of RB51-CFT positive results observed in officially Brucellosis-free (OBF) areas and, in any case, when an illegal use of RB51 vaccine is suspected, the use of the RB51-CFT alone is not sufficient to identify all the vaccinated animals. The design of a more sophisticated diagnostic protocol including an epidemiological investigation, the use of RB51-CFT, and the use of the skin test with RB51 as antigen is deemed more appropriate for the identification of RB51 vaccinated animals.

## 1. INTRODUCTION

The objective of brucellosis eradication was introduced in the Italian legislation in year 1994 [1], thus forbidding the vaccination of cattle against this disease. *Brucella abortus* strain 19 vaccine, in smooth phase, induces the production of antibodies that are detectable with the official tests and are not distinguishable from those found in infected animals. A similar problem is present with the *B. abortus* strain 45/20 vaccine, even though this strain is in rough phase [2]. *B. abortus* strain RB51 vaccine (RB51), rough mutant of the virulent strain *B. abortus* 2308, does not lead to the production of antibodies that can be detected using the conventional serological tests [3, 4]. Given the possibility of an illegal use of RB51, this characteristic may represent a serious interference in the national eradication plan and may seriously harm the achievement of officially Brucellosis-free (OBF) qualification for herds and territories, as well as the already gained qualifications. Therefore, it has been considered necessary to provide diagnostic tools able to guarantee an effective surveillance. Available literature reports the possibility to detect specific RB51 antibodies with a complement fixation test (CFT) using RB51 as antigen ([RB51-CFT]; [5, 6]). Nevertheless, the kinetics of the specific RB51 immune response in cattle after calfhood vaccination, thus the probability of identifying an illegal vaccination carried out on those animals, have never been investigated. Moreover, the RB51-CFT has never been evaluated on national cattle population. Thus, the aims of the present study are to identify the antibody kinetics of RB51-CFT in cattle vaccinated with RB51 at calfhood, to evaluate the RB51-CFT on Italian cattle population, and to carry out a preliminary study on the possibility that RB51 vaccine has been used in this population.

## 2. MATERIALS AND METHODS

### 2.1. Vaccine

The RB51 vaccine was kindly provided by CZ Veterinaria (Pontevedra, Spain), the European distributor of the product, under license from the Colorado Serum Company (Denver, Colo, USA). Once reconstituted, the vaccine contained 5 × 10^9^ CFU/mL of *B. abortus* strain RB51.

### 2.2. Animals and vaccination

Fifteen Friesian calves, aged between four to six months and obtained from OBF herds, were randomly selected and divided into two groups. One group (*n* = 10) was vaccinated subcutaneously with RB51 in accordance with the instructions of the manufacturers (2 mL reconstituted solution, containing 10 × 10^9^ colony-forming units (CFUs)). The other group (*n* = 5) was used as nonvaccinated control and the animals were inoculated subcutaneously with 2 mL sterile saline solution. All the animals were stabled with adequate space and fed with a standard diet (hay and nutritional supplements) for the entire duration of the experiment; all stages were conducted with consideration for their welfare and all procedures with animals were carried out in accordance to appropriate humane methods.

### 2.3. Serological testing and antigens

All animals were tested for anti-*B. abortus* and anti-RB51 antibodies before vaccination, on the day of vaccination (day zero) and then periodically until Day 300 postvaccination (pv). Anti-*B. abortus* antibodies were verified using the Rose Bengal test (RBT) and CFT, both performed using the *B. abortus* biovar 1 strain 99 as antigen (VLA Weybridge, UK) and according to the methods described in the 5th edition of the OIE Manual of Diagnostic Tests and Vaccines for Terrestrial Animals [7]. Anti-RB51 antibodies were monitored with an RB51 antigen-specific CFT (RB51-CFT). The antigen was prepared from the same RB51 strain used for vaccination, after growth in Brucella agar added with 5% bovine foetal serum. The antigen used in the RB51-CFT was crosstitrated with a positive serum coming from a heifer vaccinated with RB51, as described in literature [5, 6].

### 2.4. Field investigation

The field investigation covered the 2001–2004 period and was divided in two steps. For the 2001–2003 period, the investigation was carried out on sera coming from the National Serum Bank (NSB) placed in the Istituto Zooprofilattico Sperimentale dell’Abruzzo e del Molise “G. Caporale” (IZS A&M). The NSB collects and stores sera coming from cattle used as sentinel in the framework of the National Bluetongue Surveillance System (NBSS). Sentinels are bled regularly with variable frequency, depending on the season and bluetongue infection rate in the area concerned [8]. The herds to be tested were randomly selected from the sentinel herds that were present in the 12 regions in which the NBSS was in force during the period considered. Sera of all animals present in the sampled herds were tested with RB51-CFT (22,416 sera analysed from 1,948 herds, Table 1). For year 2004, sera collected by Local Veterinary Services in the framework of the National Brucellosis Eradication Plan (NBEP) were analysed by the local Istituti Zooprofilattici Sperimentali (IZS), Italian National Veterinary Laboratories. Sera were sampled according to a systematic sampling method, with a sampling interval (intended as the number of samples in between the sample selected and the next to be selected) calculated for each region on the basis of the number of samples tested for the NBEP in the year 2002. The number of serum samples expected from each region was set to 300, but not all the regions were able to reach the objective. All sera were analysed with RB51-CFT (4,559 sera analysed from 928 herds, Table 2). Moreover, NSB serum, coming from Piedmont region (2,489 sera), NBEP sera from Veneto region (300 sera), Friuli-Venezia Giulia region (185 sera), and the autonomous provinces of Trento and Bolzano (300 sera), were used to calculate the threshold of RB51-CFT (3,274 sera analysed in total).

### 2.5. Statistical analysis

In animals under experiment, and for each sampling day, mean value, 25th percentile, 75th percentile, minimum value and maximum value of titres resulting from the RB51-CFT were recorded. Sensitivity and specificity values were estimated and compared using a Bayesian approach [9]. Bayesian inference is an application of the Bayes theorem [10] that allows the investigator to integrate any previous knowledge (expressed as a prior probability distribution), with the likelihood of obtaining a certain result if the animal is infected or if the animal is healthy (likelihood functions), with the results obtained by the application of the tests to a given population (collected data). The likelihood functions depend on the sensitivity and specificity of the test(s) employed and on the uncertainty of their values. The final results are probability distribution of the number of infected animals correctly identified as infected (sensitivity) or of the number of healthy animals correctly identified as healthy (specificity) in the sample or in the population (posterior probability). Probabilities of the various possible sensitivity values were estimated using a binomial likelihood function and an uninformed Uniform(0,1) prior distribution. As existing knowledge on the sensitivity or specificity of tests was considered to be virtually nil, an uninformed Uniform(0,1) prior distribution was used. The Uniform(0,1) distribution states that prior to the collection of data, all true probability values are considered possible within the range defined for the number of true positives (sensitivity calculation) or true negatives (specificity calculation). The RB51-CFT results were expressed as the percentage of positive animals on tested; the upper and lower 95% Credibility Intervals (CI) were calculated using a beta probability distribution [11].

## 3. RESULTS

### 3.1. Serological testing on experimentally vaccinated animals

**Figure 1:**
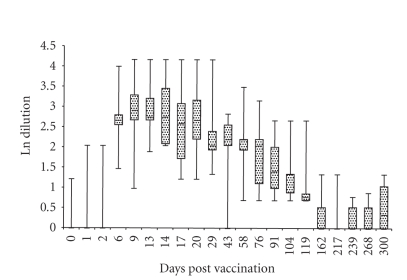
Median, maximum value, minimum value, 25th percentile, and 75th percentile of RB51-CFT results in vaccinated animals (*n* = 10).

**Figure 2:**
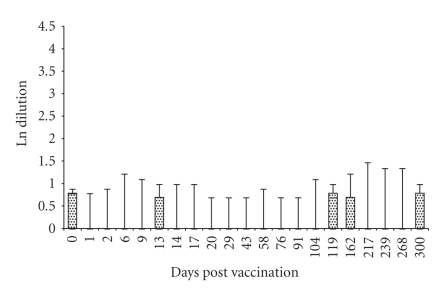
Median, maximum value, minimum value, 25th percentile, and 75th percentile of RB51-CFT results in control animals (*n* = 5).

**Figure 3:**
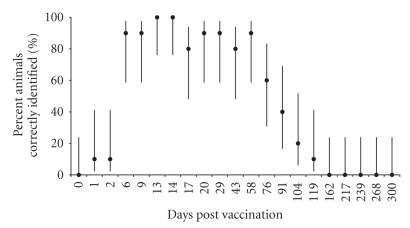
Percent vaccinated animals correctly identified by the RB51-CFT and 95% Credibility Intervals.

**Figure 4:**
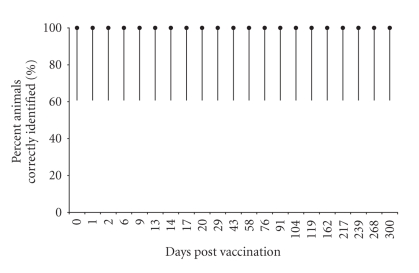
Percent control animals correctly identified by the RB51-CFT and 95% Credibility Intervals.

Sera from all animals (RB51-vaccinated and controls) gave negative results to RBT and CFT prior to vaccination, on Day zero and during the entire study. All the animals were also negative to RB51-CFT prior to vaccination and on Day zero. After vaccination, vaccinated animals developed a serological response to RB51-CFT. The results of RB51-CFT on vaccinated animals and on controls are shown in Figures 1 and 2, respectively. Using the threshold resulted from the field investigation, the percentage of animals correctly identified as vaccinated or unvaccinated by the RB51-CFT are shown in Figures 3 and 4, respectively.

### 3.2. Field investigation

**Figure 5:**
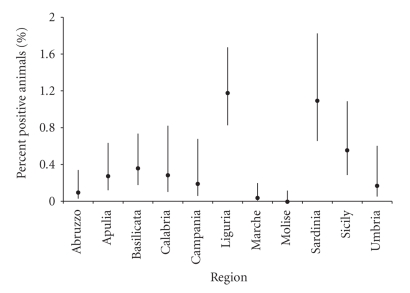
Percent of sera coming from the National Serum Bank (NSB, collected during the 2001–2003 period) and resulted positive to RB51-CFT, on regional basis.

**Figure 6:**
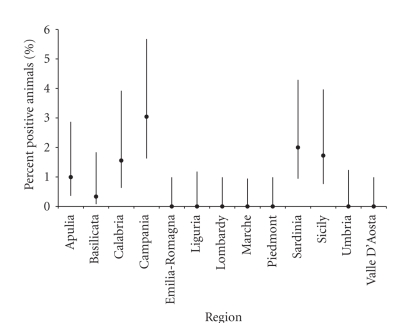
Percent of sera coming from the National Brucellosis Eradication Plan (NBEP, collected during year 2004) and resulted positive to RB51-CFT, on regional basis.

The RB51-CFT threshold has been identified in the 100% of fixation at 1 : 4 serum dilution. This result corresponds to the 90th percentile of the distribution of titres of all sera that have shown reactivity in the RB51-CFT and to the 99.9th percentile of the distribution of titres of sera coming from Piedmont, Veneto and Fiuli-Venezia Giulia regions, and the autonomous provinces of Trento and Bolzano. RB51-CFT results on NSB sera (2001–2003 period), expressed according to the region of origin and as percent of animals resulted positive on tested, are shown in Figure 5. RB51-CFT results in NBEP sera (year 2004), expressed according to the region of origin and animals resulted positive on tested, are shown in Figure 6. Given that they have been used for the threshold calculation, NSB sera from Piedmont region, as well as NBEP sera from Veneto and Fiuli-Venezia Giulia regions and the autonomous provinces of Trento and Bolzano, have not been considered in Figures 5 and 6, respectively.

## 4. DISCUSSION

The results of the present study confirm the possibility to detect specific anti-RB51 antibodies with the RB51-CFT [5, 6, 12]. Moreover, negative results to RBT and CFT confirm the impossibility to detect specific RB51 antibodies with the conventional serological tests [3, 4], while the use of Strains 19 or 45/20 would elicit the production of antibodies detectable with conventional serological tests [2]. This characteristic of RB51 might be very useful for countries having a brucellosis control program based on vaccination. Nonetheless, the choice of vaccines to be used should take into consideration also other factors, namely:

- RB51 efficiency (compared with S19) and its innocuousness remain controversial
- field experience indicates that it can induce abortion in some cases if applied to pregnant cattle and that there is excretion in milk in a relevant number of vaccinated animals
- RB51 could infect humans and it is highly resistant to rifampicin, one of the antibiotics of choice for treating human brucellosis. In addition, the diagnosis of the infection produced by RB51 requires special tests not available in most hospitals.

All these factors, together with the frequency of immunosuppressed persons in the human population should be taken into consideration in the choice of a vaccine to be used in animal populations.

Beside the availability of tests with known performances, to investigate on the possible illegal use of RB51 vaccine, it is also necessary to know the antibody kinetics in cattle vaccinated at calfhood. After showing an increase at Day 6 pv, the antibody level recorded in the 10 vaccinated animals remains practically constant for two months, and then progressively decreases (Figure 1). In particular, considering the threshold identified for the RB51-CFT with the field investigation carried out in the present study, only one animal is still correctly identified out of the 10 experimentally vaccinated at 119 days pv (Figure 3). All experimentally vaccinated animals remain negative from Day 162 pv to the end of the study (day 300 pv). The RB51-CFT shows 100% sensitivity (CI 76.2%–100%) only at days 13 and 14 pv. Control animals show results to the RB51-CFT always inferior or equal to a reaction of 100% fixation at serum dilution 1 : 4 (Figure 2). Results observed in experimentally vaccinated animals confirm the threshold identified with the field investigation and show that the possibility to use RB51-CFT for the identification of cattle vaccinated at calfhood with RB51 is limited in time. The absence of positive results in the five control animals gives an estimation of the specificity of 100%, with a CI between 60.7% and 100% (Figure 4). Nevertheless, given that this CI is somewhat wide, the RB51-CFT specificity is better defined when the results of the field investigation are considered (specificity 99.9%, CI 99.73%–99.96%). The field investigation reveals that in some Italian regions (namely, Basilicata, Calabria, Campania, Liguria, Apulia, Sardinia, Sicily, and Umbria) the number of positive results (Figure 5) is higher than what would be expected on the basis of the RB51-CFT specificity (as it has been standardized). As a matter of fact, the expected percentage of false-positive results to the RB51-CFT would be 0.1%, with a lower CI of 0.04%. Moreover, in the case of Liguria, Sicily, and Sardinia regions, the lower CI of the percent positive animals is higher than the upper CI of the percent positive animals that would be expected on the basis of the test specificity. Therefore, it is unlikely that the frequency of positive results observed in these latter regions would be only due to the effect of case. The results shown in Figure 5 (sera from the 2001–2003 period) are confirmed for six regions (namely, Basilicata, Calabria, Campania, Apulia, Sicily, and Sardinia) by the results observed on samples collected during 2004 (Figure 6). These regions, in year 2004 also, show a percentage of positive animals higher than expected on the basis of test specificity. The positive results observed on sera collected during the 2001–2003 period from Umbria and Liguria regions (Figure 5) are not confirmed on sera collected in year 2004, where no positive result was recorded. Moreover, for some regions (namely, Calabria, Campania, Apulia, Sicily, and Sardinia) the lower CI of the percentage of positive animals is higher than the percentage expected on the basis of the upper CI of test specificity (Figure 6). Although a possible illegal use of RB51 vaccine could be explained for some of these regions (Calabria, Campania, Apulia, Sicily) by the high brucellosis prevalence, by the difficulties encountered in the eradication process, and by the frequent reinfections of cleaned herds (as detected during the NBEP, Italian Ministry of Health, personal communication), an illegal use of this vaccine remains difficult to explain in Sardinia region, which reached the status of OBF region in April 2003 [13]. Nevertheless, Sardinia has intensive animal trade with other Italian regions that in several occasions was responsible of the reintroduction of Brucellosis in the island. In the case of Sardinia, RB51-vaccinated animals could have been imported from other Italian regions and this could explain the presence of a positivity rate significantly higher than that expected on the basis of the test specificity.

Given the nature of the disease (vaccination cannot avoid the infection of the animal and the related carrier state), the passage from a brucellosis control program to a brucellosis eradication program would necessarily imply to forbid the use of any kind of vaccination. Once vaccination is forbidden, an illegal use of RB51 in cattle can be expected in areas with a relatively high prevalence because farmers would try to decrease the number of abortions (and the other economic losses associated with brucellosis, such as the drop in fertility and/or in milk production) by using a tool that cannot be identified by the Veterinary Services with the routine brucellosis diagnostic procedures. Lacks in the Official Veterinary Services inspection activities, together with the absence of an effective surveillance system, are conditions that encourage the illegal vaccination practices in those areas. The uncontrolled spread of the vaccinal strain would have effects on the epidemiology of the field strain also, and this cannot be properly assessed (and therefore controlled) without a reliable diagnostic tool to identify animals vaccinated with RB51.

The results of the present study suggest that in case of RB51-CFT-positive results observed in OBF areas (as the ones observed in Sardinia region) and, in any case, when an illegal use of RB51 vaccine is suspected, the use of the RB51-CFT alone is not sufficient but it would be appropriate to design a more sophisticated diagnostic protocol. The use of a protocol which would include an epidemiological investigation, the use of RB51-CFT, and the use of the skin test with RB51 as antigen [12] could give important elements to the final correct interpretation of RB51-CFT results and identification of RB51-vaccinated animals.

## Figures and Tables

**Table 1:** Number of sera from the National Serum Bank (NSB, collected during the 2001–2003 period) and tested with RB51-CFT, on regional basis.

Region of origin	No. of sera tested	No. of herds tested
Abruzzo	2,123	177
Apulia	1,836	207
Basilicata	1,957	222
Calabria	1,064	111
Campania	1,062	117
Liguria	2,552	193
Marche	2,825	238
Molise	2,584	217
Piedmont	2,489	139
Sardinia	1,282	110
Sicily	1,446	114
Umbria	1,196	103

Total	22,416	1,948

**Table 2:** Number of sera from the National Brucellosis Eradication Program (NBEP, collected during year 2004) and tested with RB51-CFT, on regional basis.

Region of origin	No. of sera tested	No. of herds tested
Apulia	302	61
Basilicata	300	59
Calabria	266	54
Campania	300	59
Emilia-Romagna	300	60
Friuli-Venezia Giulia	185	38
Liguria	251	55
Lombardy	300	60
Marche	315	79
Piedmont	300	61
Sardinia	300	62
Sicily	300	57
Trentino-Alto Adige	300	61
Umbria	240	46
Valle d'Aosta	300	56
Veneto	300	60

Total	4,559	928
